# Microvascular reactivity in women with gestational diabetes mellitus studied during pregnancy

**DOI:** 10.1186/s13098-015-0017-4

**Published:** 2015-03-28

**Authors:** Isabelle EA Pontes, Karine F Afra, José R Silva, Paulo SN Borges, Geraldine F Clough, João GB Alves

**Affiliations:** Mother and Child Unit, Instituto de Medicina Integral Prof. Fernando Figueira (IMIP), Rua dos Coelhos, 300, 50070-550 Boa Vista Recife, Pernambuco Brazil; Faculty of Medicine, University of Southampton, Southampton, UK

**Keywords:** Microcirculation, Endothelium function, Pregnancy, Gestational diabetes

## Abstract

**Aim:**

To compare microvascular reactivity assessed in the skin using laser Doppler fluximetry (LDF) in women with gestational diabetes mellitus (GDM) and gestational age-matched control during pregnancy.

**Methods:**

110 pregnant women at ~33 weeks gestation participated in the study. Skin microvascular reactivity was evaluated by LDF, at rest, during the response to brief arterial occlusion (post occlusive hyperaemic response) and during sympathetically mediated vasoconstrictor response to deep inspiratory breath hold.

**Results:**

No statistically significant differences were found in the microvascular variables studied (resting and maximum rate flux, post-ischaemic reactive hyperaemia and deep inspiratory breath holds) between +GDM and –GDM groups women. In women with GDM there was a negative correlation between resting flux and the response to the oral glucose tolerance test (OGTT), r = -0.282 (p = 0.037). There was also a negative correlation between the response to the OGTT and the sympathetically mediated constrictor response to inspiratory breath holds (r = -.298, p = .030) but not in women with GDM (r = .102, r = .468).

**Conclusion:**

Attenuated microvascular reactivity as an early marker of endothelial dysfunction is not present in women with GDM when assessed during pregnancy.

## Introduction

Concurrent with the rising prevalence of obesity, especially among women of reproductive age, the prevalence of gestational diabetes mellitus (GDM) is increasing and has been a health concern worldwide [1]. GDM is associated with adverse perinatal outcomes for both mother and fetus [2]. GDM women and their offspring are more likely to be obese and have insulin resistance throughout life [3]. Women with a history of GDM have a 70% higher incidence of cardiovascular disease (CVD) as compared to their peers and even milder gestational impaired glucose tolerance has been associated with an enhanced cardiovascular risk factor profile and subsequent type 2 diabetes [4,5].

Endothelium dysfunction has been indicated as an early event in the development of CVD and the main underlying mechanism of type 2 diabetes complications in the peripheral and microvasculature [6]. Microvascular complications represent the main cause of morbidity and mortality in diabetes mellitus. Microvascular abnormalities and impaired tissue perfusion are associated with insulin resistance and may precede the clinical manifestations of diabetic microvascular disease and macrovascular dysfunction [7]. Endothelial dysfunction has been showed as an early and accurate predictor of long-term cardiovascular events in patients with diabetes [8].

Impaired endothelium-dependent relaxation in larger blood vessels has been observed in women with a history of GDM assessed months or years after delivery in some but not all studies and these findings remain controversial [9-12]. Similarly, impaired microvascular responses have also been reported in women with previous GDM studied some years after pregnancy [11,13] suggesting that abnormal microvascular function could represent a novel mechanism contributing to the elevated risk of CVD in these women. However, microvascular responses have yet to be fully evaluated during pregnancy in women newly diagnosed with GDM.

Laser Doppler flowmetry (LDF) is a non-invasive and reproducible method with which to investigate microvascular function in the skin [14,15]. It has been widely used in a clinical setting combined with provocation tests to investigate vascular mechanism in hypertension [16], obesity [17] and in diabetes [18]. Several studies have shown skin microvascular reactivity to be attenuated in insulin resistant individuals and in individuals with type 1 and type 2 diabetes compared with control [14,17,19].

This study aims to compare microvascular reactivity assessed in the skin using LDF in women with GDM and gestational age-matched controls during pregnancy and to explore the association between microvascular reactivity and other CVD risk factors in these women.

## Material and methods

A cross-sectional study enrolled 110 pregnant women, 55 with GDM diagnostic and 55 normoglycemic. All study participants were recruited from the prenatal care of Instituto de Medicina Integral Prof. Fernando Figueira (IMIP) from September 2013 to August 2014. This project was previously approved by the Ethics Committee (n°: 02345112.6.0000.5201) in Research of IMIP and all pregnant women signed an informed consent form.

GDM diagnosis was based on the *International Association of Diabetes and Pregnancy Study Group* [20] criteria. A glucose tolerance test (OGTT) was performed between 24^th^ and 28^th^ gestational week with a 75 g of dextrose; a fasting glucose ≥ 92 mg/dl, or 1 h post-test ≥180 mg/dl or 2 h post-test ≥ 153 mg/dl were all considered as gestational diabetic criteria. Pregnant women with previous history of GDM, type 1(T1D) or type 2 diabetes (T2D), hypertensive disorder and mental or neurologic disease were excluded. Anthropometric and obstetrical variables were collected by an interviewer.

Body mass index (BMI) was based on the Atalah et al. [21] curve for gestational week. The gestational age was derived from the last menstrual period, otherwise, gestational age was corrected on the basis of ultrasonographic measurements.

Skin microvascular reactivity was evaluated by LDF at approximately 28 weeks gestation using a 785 nm, 1 mW low power red laser light source (VMS-Moor Instruments, UK). Single point laser Doppler flow probes (DP1T, Moor, Instruments, UK) were attached to the skin using double-sided sticky O-rings, one on the volar surface of the left forearm approximately 10 cm from the wrist and avoiding visible veins, and one on the pulp of the middle finger of the same arm.

All tests were conducted after a period of acclimatization in a temperature-controlled room (23°C ± 1°C), with the participants sitting comfortably with their arms resting at heart level and legs elevated. Participants were instructed not to take exercise or to consume caffeinated drinks in the 24 hours that preceded the evaluation. All tests were performed by the same researcher.

**Figure 1:**
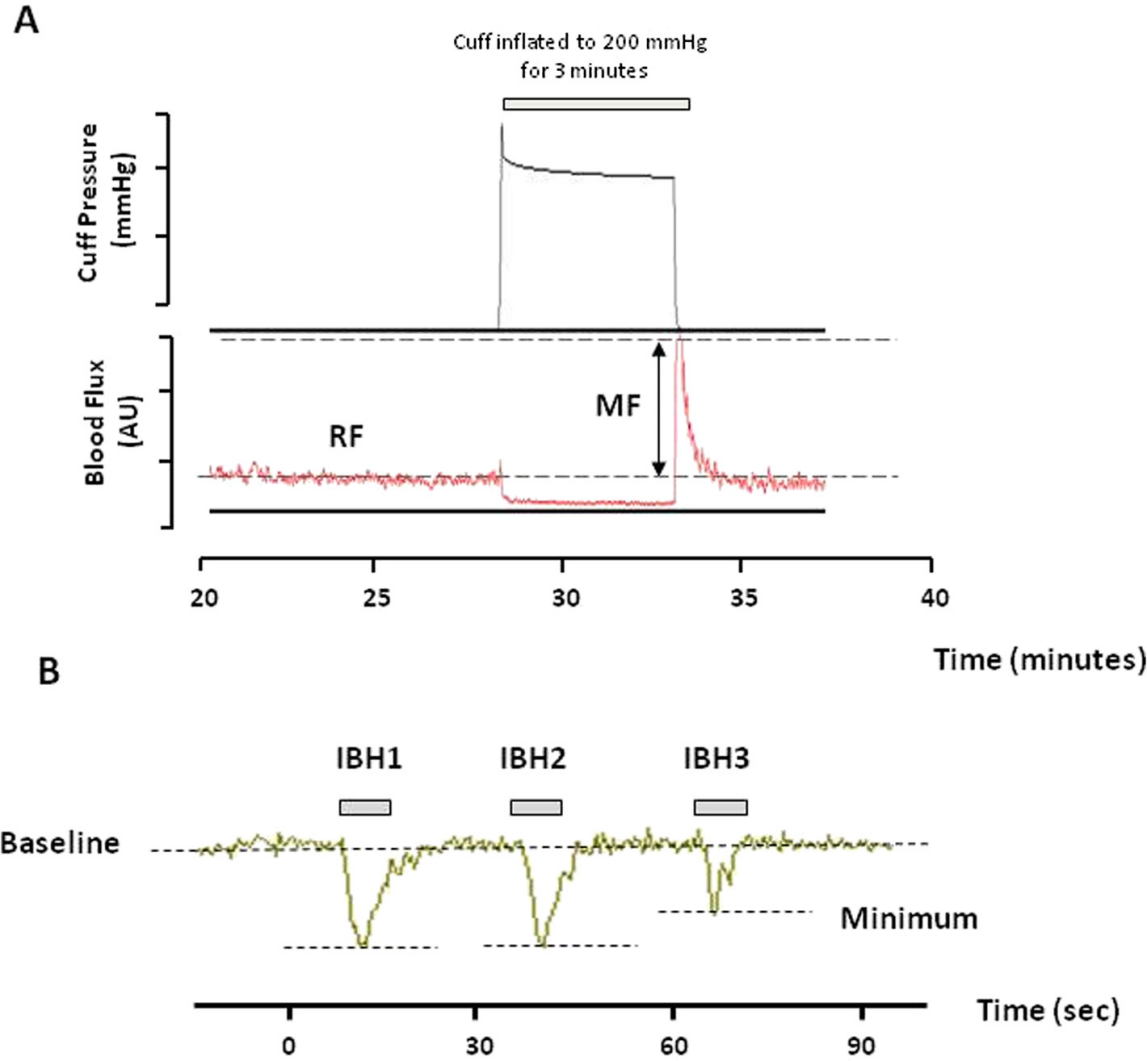
**Representative recording of blood flux from the skin of the forearm and finger measured using laser Doppler fluximetry. A**. RF = resting blood flux, MF = maximum flux on release of arterial occlusion at forearm (200 mmHg for 3 min). **B**. Baeline and minimum flux measured at the finger over the last 3 sec of three 6 second inspiratory breath holds.

Baseline skin perfusion flux was continuously recorded for up to 10 min prior to perturbation of skin blood flow. Skin microvascular reactivity was assessed (a) during and for 5 min after the hyperemic response to brief arterial occlusion (200 mmHg for 3 min) using a pressure cuff placed around the upper arm (VMSPRES Moor Instruments, UK) [15], and (b) in response to transient sympathetic nervous system-mediated vasoconstriction to deep inspiratory breath hold (IBH) (3 × 6 second deep inspiratory breath holds with 1 min between each) [22] (Figure 1). Data were stored for later analysis using the manufacturer’s software.

The variables analyzed to evaluate the microvascular dilator capacity at the forearm were resting flux (RF, in arbitrary perfusion units PU) measured as the average flux over the 5 min before cuff inflation; maximum flux (MF, PU) during the post occlusive hyperemic (PORH) response; and the ratio of MF to RF (Figure 1A). The area under the hyperemia response was also estimated. The vasoconstrictor response to IBH was taken from the flux recorded at the finger as the minimum value over the last 3 seconds of the breath hold and expressed as % change from baseline of the mean minimum response for the three breath holds [22] (Figure 1B).

Data analysis was performed using SPSS 13.0 for Windows. Measures of central tendency and dispersion for numeric variables were obtained. Data were tested for normality using a Kolmogorov-Smirnov test for quantitative variables. Data are reported as means and standard deviations (mean ± SD) for normally distributed variables, or as median and interquartile range (IQR) for non-normally distributed variables. Comparison of measurements in women with and without GDM for continuous variables was undertaken by paired t-tests or Mann-Whitney test. Pearson and Spearman rank correlation coefficients were used to investigate associations between normally and non-normally distributed variables, respectively. In all cases a value of p < 0.05 was taken to indicate significance.

## Results

198 pregnant women were enrolled in the study and 144 were eligible; 54 were excluded by blood hypertension, type 1 or type 2 diabetes or twin pregnancy. From the eligible pregnant women, 34 did not agree to participate. The final sample consisted of 110 pregnant women, 55 with GDM and 55 controls. Among the 55 GDM pregnant women, 4 were taking medication for hyperglycemia control and 51 were controlled with an adequate diet.

Women were studied during week 33 ± 4 of pregnancy. The women had an average age of 27.5 ± 6 years and BMI at the time of evaluation of 29 ± 4 kg/m^2^. Across the whole cohort, BMI at the time of study was positively correlated with systolic (r = 0.261, p = 0.006) and diastolic (r = 0.239, p = 0.012) blood pressure and with fasting glucose (r = 0.213, p = 0.026). Age was positively correlated with the response to the OGTT at both 1 h (r = 0.417, p < 0.001) and 2 h (r = 0.316, p < 0.001).

Table 1 summarizes the anthropometric and obstetric characteristics of the two groups of women with and without GDM. Women with GDM were significantly older with a higher BMI than women without GDM. Primiparous prevalence showed no difference among women with and without GDM and was 54.5% and 57.4%, respectively.

**Table 1:** **Anthropometric and obstetric variables of pregnant women with and without gestational diabetes mellitus (GDM)**

	**Group**		
**Variables**	**+GDM (n = 55)**	**–GDM (n = 55)**	**p-value**
Age (years)	29.5 ± 6.5	25.6 ± 4.7	<0.001^a^
Gestational age at study	33.2 (3.7)	32.9 (3.8)	0.550^b^
Height (cm)	160.6 (7.6)	157.9 (7.2)	0.312^b^
Weight before pregnancy (kg)	68.0 ± 15.7	63.5 ± 9.6	0.072^a^
Weight at test (kg)	77.8 ± 15.0	73.9 ± 9.7	0.114^a^
Weight gain (kg)	9.8 ± 5.2	10.0 ± 4.4	0.772^a^
BMI before pregnancy (kg/m^2^)	26.2 ± 4.9	24.2 ± 3.3	0.014^a^
BMI at test (kg/m^2^)	30.0 ± 4.6	28.2 ± 3.2	0.017^a^
Fasting plasma glucose (mg/dl)	95.8 ± 18.9	81.1 ± 6.2	<0.001^a^
Plasma glucose after 1 h OGGT (mg/dl)	166.4 ± 30.3	125.2 ± 18.5	<0.001^a^
Plasma glucose after 2 h OGTT (mg/dl)	156.1 ± 33.2	109.5 ± 17.4	<0.001^a^
Systolic blood pressure (mmHg)	104.4 (10.3)	106.8 (7.2)	0.186^b^
Diastolic blood pressure (mmHg)	67.0 (9.2)	67.9 (6.8)	0.616^b^

Date are mean ± SD or median(IQR) ^a^Student t Test ^b^Test Mann-Whitney test.

**Figure 2:**
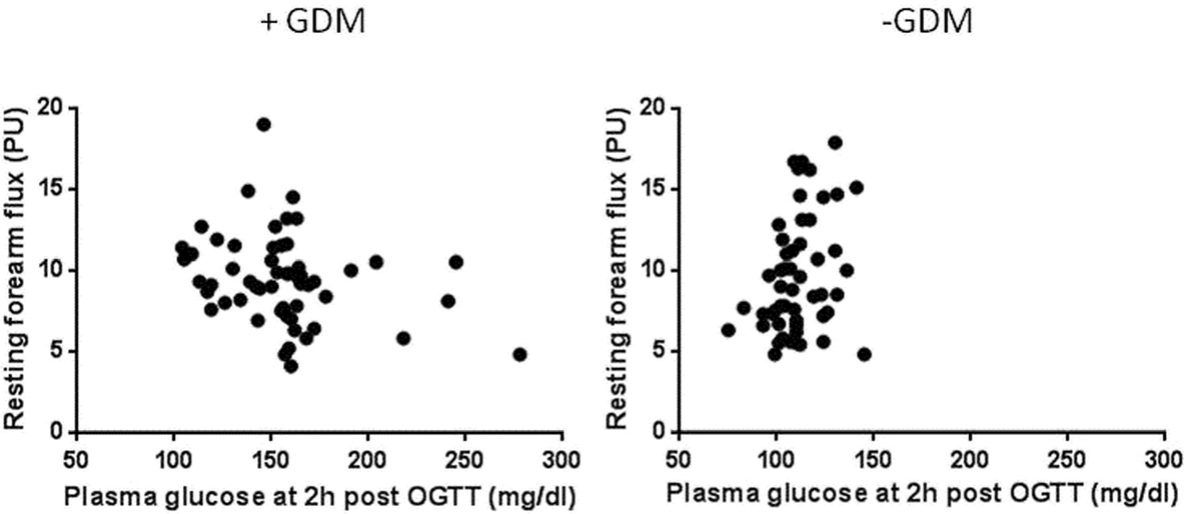
**Scatter plots showing the relationship between resting forearm blood flux measured using laser Doppler fluximetry and glycaemic response to an oral glucose tolerance test (OGTT) measured at 2 hours in women with and without gestational diabetes mellitus (GDM) performed between 24th and 28th gestational week.** +GDM r = -0.282, p = 0.037; –GDM r = 0.296, p = 0.030.

No statistically significant differences were found in the microvascular variables studied at the forearm (RF, MF, MF/RF, area under the hyperaemic response curve) between +GDM and –GDM groups (Table 2). There was a negative correlation between resting forearm flux and the response to the OGTT at 2 h (r = -0.282, p = 0.037) women with GDM (Figure 2). In women without GDM the response to the OGTT and resting forearm blood flux was positively correlated (r = 0.296, p = 0.030). Baseline flux measured at the finger was lower in women with GDM (p = 0.026) and correlated with weight gain during pregnancy (r = 0.330, p = 0.014). There was also a negative correlation between the response to the OGTT at 1 h and the sympathetically mediated constrictor response to IBH (%IBH) (r = -0.298, p = 0.030) in women without GDM but not in women with GDM (r = 0.102, r = 0.468).

**Table 2:** **Skin blood flux measured by laser Doppler fluximetry in women with and without gestational diabetes mellitus GDM (n = 55 per group)**

	**Group**		
**Variables**	**+GDM**	**–GDM**	**p-value**
Resting Forearm Flux (RF, PU)	9.7 ± 2.7	9.6 ± 3.5	0.779^a^
Maximum Forearm Flux (MF, PU)	64.2 ± 23.9	64.8 ± 36.1	0.917^a^
MF/RF	6.9 (2.5)	6.9 (2.9)	0.540^b^
Area of Hyperemia (PU.s)	14450 ± 718	1293 ± 824	0.297^a^
Baseline Finger Flux (PU)	279.1 ± 78.8	323.6 ± 96.7	0.026^a^
Minimum IBH Finger flux (PU)	138.4 ± 59.1	158.8 ± 85.5	0.152^a^
IBH change from baseline (%)	48.8 ± 20.8	49.4 ± 27.3	0.896^a^

Data are mean ± SD or median(IQR) ^a^Student t test ^b^Mann-Whitney test.

Values were obtained before and during the reactive hyperaemic response to arterial occlusion at the forearm and deep inspiratory breath hold (IBH) at the finger.

## Discussion

In this study we found no differences in microvascular reactivity between women with and without GDM when assessed during pregnancy. In women without GDM glucose homeostasis was associated positively with resting skin blood flux and negatively with sympathetically mediated vascular tone, both of which adaptive responses were lost in women with GDM. In women with GDM we found a negative correlation between resting skin microvascular blood flux and glucose homeostasis. Across the whole study cohort there was a relationship between glucose homeostasis and age, pregnancy BMI and weight gain, but not microvascular status. Together these data suggest that there is no impairment in microvascular reactivity in the skin of women recently diagnosed with GDM and studied in the 3^rd^ trimester.

Previous studies have shown that in women with a history of gestational diabetes endothelial dysfunction is present in forearm conduit arteries [10,11,13] and that these women have a high risk of developing vascular disease and type 2 diabetes, despite normalization of hyperglycemia after delivery [23]. Most studies that have explored endothelium function in women with GDM have assessed vascular function at the level of the macrovasculature months to years after delivery. Vascular dysfunction seen in the early post-partum period in previously GDM women has been taken to be indicative of the potential for short term exposure to hyperglycaemia to have long-term consequences on cardiometabolic health [13]. However, it remains uncertain whether endothelial dysfunction is present at the time of diagnosis of GDM or whether early attenuation in microvascular perfusion capacity may be contributory to later risk of developing hypertension and T2D. Indeed endothelium dysfunction has been detected in the fetoplacental circulation in GDM [24]. Thus it is possible that very early deficits in the microvasculature may contribute to poor pregnancy outcomes.

To our knowledge this is the first study in which microvascular reactivity, investigated as both dilator and constrictor responses in the skin, has been evaluated in women with GDM during pregnancy. We observed a negative correlation between resting skin blood flow and glycaemic control (plasma glucose levels at 1 and 2 h post-test). This finding is consistent with that of Mrizak et al [24] who showed a negative correlation between insulin resistance (HOMA-IR) and microvascular reactivity; and supportive of a pathophysiological link between GDM, type 2 diabetes and metabolic syndrome.

Our data indicate that when assessed during pregnancy, neither resting skin microvascular blood flow nor dilator capacity to a short ischemic insult differed in women matched for gestational age, with and without GDM. Our finding of a lack of difference in vascular reactivity between non-obese women with and without GDM is consistent with those of Brewster et al [12] and Hannemann et al [11] who found no evidence of endothelial dysfunction assessed in the brachial artery using flow mediated dilation (FMD) in women with previous GDM studied 1-10 y postpartum. Davenport et al [13] conversely found decreased endothelial function assessed by FMD in women eight weeks after delivery. Our findings in the microvasculature of a lack of difference between women with and without GDM contrast with those of Hannemann et al. [11] who in the same group of women in which they report no different in FMD, studied up to 10 years after pregnancy showed impairment of the maximal hyperaemia to local warming in the skin microvasculature. They also contrast with Hu et al [25] who reported a reduced cutaneous ACh-mediated dilation in .the hands and feet of asymptomatic women with a history of GDM, 2 to 4 years after pregnancy. While some of these differences may be due to the part of the vascular tree studied, brachial artery vs microcirculation, the differences observed within the same vascular bed (the skin microvasculature) albeit of the forearm, hands and feet, are less easily resolved. It may be speculated that when studied at the time of diagnosis there has been insufficient time for the hyperglycaemic state to impact on endothelial function. The attenuated vascular responses and endothelial dysfunction seen months or years after a GDM pregnancy may be a consequence of the persistence of clinical and/or subclinical hyperglycemia after delivery and the subsequent development of vascular complications that predispose to increased CVD risk and the development of NIDDM.

Factors additional to dysglycaemia during pregnancy may also contribute to later endothelial dysfunction. These include an increased inflammatory state [26] demonstrated to be negatively correlated with skin blood flow in women with GDM [27]. These authors report an attenuated endothelium-dependent ACh-mediated vasodilation measured in the skin of the forearm using LDF, in no-obese GDM women, studied similarly to us in the third trimester of pregnancy. An altered PORH response has also been reported in pregnant hypercholesterolaemic patients compared with age and gestational age-matched controls [28]. We were unable to measure markers of inflammatory status or cholesterol in our cohort, but cannot rule out the possibility that these factors may contribute to altered microvascular reactivity in GDM.

Pregnancy is associated with positive cardiovascular adaptations including a decreased total peripheral resistance (TPR) designed to promote the convective delivery of oxygen and nutrients to the developing fetus [29]. The values of resting skin blood flux and the changes in microvascular flux during PORH and IBH are consistent with those reported previously by us and others in similarly aged cohorts [13,14] and there was no correlation with blood pressure. It is possible however that the differing direction of the associations between glycaemic control and sympathetically-mediated vasoconstrictor response in the two groups of women may be contributory to the development of dysglycaemia and GDM in some women. There are considerations that must be taken into account when interpreting these data. Current BMI, pre-pregnancy BMI and age were higher in women with GDM, all of which are recognized risk factors for GDM [30]. The mean age of the +GDM group was also 4 years greater than –GDM. Altered microvascular reactivity has been shown to be associated with metabolic phenotype and with age [14]. Thus it is surprising that we did not see differences in any of our microvascular measures between the two groups. Glucose levels were being managed in the majority of our GDM patients by diet or medicines which may contribute to the lack of difference in microvascular reactivity between the two study groups. We did not measure insulin or lipoproteins profile which have been described as associated with endothelium function. The study was a cross-sectional study design and we were unable to explore the progression of changes in microvascular reactivity post partum.

## Conclusion

We concluded that microvascular reactivity is unaltered in women with GDM when assessed during pregnancy. Thus despite changes in endothelial function that are seen postpartum in women with previous gestational dysglycaemia and their long term cardiovascular risk, it is unlikely that early changes in microvascular function contribute to this. Additional factors such as maternal obesity and weight gain during pregnancy that are associated with microvascular dysfunction across the study cohort may play a greater role in predisposing to later risk of developing vascular disease and type 2 diabetes.
